# Fast permutation preconditioning for fractional diffusion equations

**DOI:** 10.1186/s40064-016-2766-4

**Published:** 2016-07-19

**Authors:** Sheng-Feng Wang, Ting-Zhu Huang, Xian-Ming Gu, Wei-Hua Luo

**Affiliations:** School of Mathematical Sciences, University of Electronic Science and Technology of China, Chengdu, 611731 Sichuan People’s Republic of China; Data Recovery Key Laboratory of Sichuan Province, Neijiang Normal University, Neijiang, 641100 Sichuan People’s Republic of China

**Keywords:** Fractional diffusion equations, Shifted Grünwald formula, Toeplitz matrix, BiCGT method, BiCRT method, Fast Fourier transforms, 65F10, 65L12, 65L20, 65T50, 26A33

## Abstract

In this paper, an implicit finite difference scheme with the shifted Grünwald formula, which is unconditionally stable, is used to discretize the fractional diffusion equations with constant diffusion coefficients. The coefficient matrix possesses the Toeplitz structure and the fast Toeplitz matrix-vector product can be utilized to reduce the computational complexity from ${\mathcal {O}}{(N^{3})}$ to ${\mathcal {O}}{(N \log N)}$, where *N* is the number of grid points. Two preconditioned iterative methods, named bi-conjugate gradient method for Toeplitz matrix and bi-conjugate residual method for Toeplitz matrix, are proposed to solve the relevant discretized systems. Finally, numerical experiments are reported to show the effectiveness of our preconditioners.

## Background

In the last few decades, many anomalous diffusion phenomena have been found in the real world, which lead to the generation of the fractional diffusion equations (FDEs). The FDEs emerge from numerous research fields such as modeling chaotic dynamics of classical conservative systems (Zaslavsky et al. 1993), groundwater contaminant transport (Benson et al. 2000a, b), turbulent flow (Carreras et al. 2001; Shlesinger et al. 1987), and applications in physics (Sokolov et al. 2002), finance (Raberto et al. 2002), biology (Magin 2006), hydrology (Baeumer et al. 2001) and image processing (Blackledge 2009; Bai and Feng 2007). Usually, it is unavailable to access the closed-form analytical solutions of the FDEs (Alquran et al. 2015; Allan and Al-Khaled 2006; Sababheh et al. 2003). Therefore, many numerical approaches for the FDEs have been proposed and developed intensively in the last decade, for instance Zhang et al. (2010), Ervin et al. (2007), Langlands and Henry (2005), Liu et al. (2004), Meerschaert and Tadjeran (2004, 2006), Tian et al. (2015), Gu et al. (2015). However, even if the discretized approach of the FDEs is implicit, it still can result in unconditionally unstable (Meerschaert and Tadjeran 2004, 2006) because of the nonlocality of the fractional differential operators.

In order to overcome the difficulty of the stability, (Meerschaert and Tadjeran 2004, 2006) put forward a shifted Grünwald discretization to approximate FDEs with a left-sided fractional derivative and the FDEs with two-sided fractional derivatives, respectively, and their method has been proved to be unconditionally stable. However, it is worth noting that most of the numerical methods for FDEs tend to generate full coefficient matrices, which require computational cost of ${\mathcal {O}}({N}^{3})$ and storage of ${\mathcal {O}}({N}^{2})$, there is no doubt that it will certainly increase the computational work; see, e.g., Wang et al. (2010) for a discussion of these issues.

Recently, there is some progress on fast numerical solutions of FDEs. Wang et al. (2010) discovered that the full coefficient matrix generated by Meerschaert and Tadjeran (2006) method has a good feature, i.e., it can be written as a sum of diagonal-multiply-Toeplitz matrices. Thus the storage requirement is reduced from ${\mathcal {O}}({N}^{2})$ to ${\mathcal {O}}(N)$. As we know, the Toeplitz matrix-vector product (MVP) can be computed in ${\mathcal {O}}(N \log N)$ operations by the fast Fourier transforms (FFTs) (Chan and Ng 1996). Fast methods Lei and Sun (2013), Popolizio (2015), Gu et al. (2015, 2015) have been developed to solve FDEs with the shifted Grünwald formula. Wang and Wang (2011) proposed a conjugate gradient normal residual (CGNR) to solve the discretized system by Meerschact and Tadjeran’s method with the computational cost of ${\mathcal {O}}{(N \log N)}$. The preconditioned CGNR with a circulant preconditioner is proposed by Lei and Sun (2013), to solve FDEs by Meerschact and Tadjeran’s method with constant diffusion coefficients.

In this paper, we employ the implicit finite difference method to discretize the FDEs and the problem is transformed to solve a linear nonsymmetric Toeplitz system in each time step. Since, the Bi-Conjugate Gradient (BiCG) (Saad 2003, pp. 234–236) and Bi-Conjugate Residual (BiCR) (Sogabe et al. 2009) can be regarded as two effective methods for solving the nonsymmetric system. There is no doubt that the two methods with Toeplitz fast MVP can be used to solve such discretized Toeplitz linear systems. However, from Sogabe et al. (2005), Pestana and Wathen (2015), if the two iterative methods are employed directly, then we indeed fail to make full use of Toeplitz structure of the discretized system, it also means that their computational cost fail to attain optimality. Hence, it is still worth finding more effective methods to reduce the computational complexity. Recently, in Sogabe et al. (2005), Pestana and Wathen (2015), a permutation matrix *P* was introduced to transform the nonsymmetric matrix into a symmetric one so as to improve the performance of iterative methods. In view of this point, we re-explain the ideas in Sogabe et al. (2005), Pestana and Wathen (2015) as a kind of preconditioning techniques for solving the discretized system of the FDEs by the method of Meerschaet and Tadjeran. More precisely, we do equivalent transformation for the original discretized system, left multiplying by a permutation matrix (Pestana and Wathen 2015) at the same time, then we obtain a new symmetric linear system with the coefficient matrix being a Hankle matrix, which has the same solution with the original discretized system. As we know, the symmetric linear systems are usually simpler to be solved than the nonsymmetric cases. Conjugate Gradient (CG) and Conjugate Residual (CR) are two effective methods for solving symmetric linear system. In this paper, we extend CG and CR to BiCGT and BiCRT, respectively, which are proposed to solve the equivalent equation. The numerical results show that both BiCGT and BiCRT are more competitive than CGNR.

The paper is organized as follows. in Sect. 2, we briefly introduce the discretization of FDEs by finite difference method. In Sect. 3, we construct the permutation preconditioner and propose BiCGT and BiCRT to solve the equivalent system of linear equations. In Sect. 4, numerical results are reported to illustrate the efficiency of the proposed methods. Concluding remarks are given in Sect. 5.

## Discretization of FDEs by finite difference method

In this section, we are interested in solving an initial-boundary value problem of the following FDEs,

1 $${\left\{ \begin{array}{ll} \frac{\partial u(x,t)}{\partial t}=d_{1}\frac{\partial ^{\alpha }u(x,t)}{\partial _{+}x^{\alpha }}+ d_{2}\frac{\partial ^{\alpha }u(x,t)}{\partial _{-}x^{\alpha }}+f(x,t),\quad x\in (x_L, x_R),~ t\in (0,T],\\ u(x_L,t)=u(x_R,t)=0,\quad 0\le t\le T,\\ u(x,0)=u_0(x),\quad x\in [x_L,x_R],\\ \end{array}\right.}$$

where $\alpha \in (1,2)$ is the order of the space fractional derivative, *f*(*x*, *t*) is the source term, and the diffusion coefficients satisfying $d_{1}\ge 0$, $d_{2}\ge 0$, and $d_{1}+d_{2}\ne 0$. In this paper, we use the Grünwald-Letnikov form Podlubny (1999) to define the left-sided and the right-sided fractional derivatives $\frac{\partial ^{\alpha }u(x,t)}{\partial _{+}x^{\alpha }}$ and $\frac{\partial ^{\alpha }u(x,t)}{\partial _{-}x^{\alpha }}$:

$$\begin{aligned} \frac{\partial ^{\alpha }u(x,t)}{\partial _{+}x^{\alpha }}&= \lim _{{\varDelta }x\rightarrow 0^{+}} \frac{1}{{\varDelta }x^{\alpha }}\sum _{k=0}^{\lfloor (x-x_{L})/{\varDelta }x \rfloor }g_{k}^{(\alpha )}u(x-k{\varDelta }x,t),\\ \frac{\partial ^{\alpha }u(x,t)}{\partial _{-}x^{\alpha }}&=\lim _{{\varDelta }x\rightarrow 0^{+}} \frac{1}{{\varDelta }x^{\alpha }}\sum _{k=0}^{\lfloor (x_{R}-x)/{\varDelta }x \rfloor }g_{k}^{(\alpha )}u(x+k{\varDelta }x,t), \end{aligned}$$

where $\lfloor \cdot \rfloor$ denotes the floor function, and the Grünwald coefficients $g_{k}^{(\alpha )}$ are defined as follows

2 $${\left\{ \begin{array}{ll} g_{0}^{(\alpha )}&=1,\\ g_{k}^{(\alpha )}&=\frac{(-1)^k}{k!}\alpha (\alpha -1)\cdots (\alpha -k+1),\quad k=1,2,3,\ldots , \end{array}\right. }$$

which can be evaluated by the recurrence relation

$$g_{k+1}^{(\alpha )}=\left(1-\frac{\alpha +1}{k+1}\right)g_{k}^{(\alpha)},\quad k=0,1,2,\ldots.$$

In order to derive the proposed scheme, let $h=\frac{{x}_{R}-{x}_{L}}{N+1}$ and ${\varDelta }{t}=T/M$ be the sizes of spatial grid and time step, respectively (*N*, *M* are positive integers). Define ${x}_{i}={x}_{L}+ih$ ($i=0,1,\ldots ,N+1$) and the temporal partition ${t}_{m}=m{\varDelta }{t}$ ($m=0,1,\ldots ,M$). Let ${u}_{i}^{(m)}=u({x}_{i},{t}_{m})$, ${f}_{i}^{(m)}=f({x}_{i},{t}_{m})$. We employ the shifted Grünwald approximation (Meerschaert and Tadjeran 2004, 2006):

$$\begin{aligned} \frac{\partial ^{\alpha }u({x}_{i},{t}_{m})}{\partial _{+}x^{\alpha }}&= \frac{1}{h^{\alpha }}\sum _{k=0}^{i+1}g_{k}^{(\alpha )}u_{i-k+1}^{(m)} +{\mathcal {O}}{(h)},\;\\ \frac{\partial ^{\alpha }u({x}_{i},{t}_{m})}{\partial _{-}x^{\alpha }}&= \frac{1}{h^{\alpha }}\sum _{k=0}^{N-i+2}g_{k}^{(\alpha )}u_{i+k-1}^{(m)} +\mathcal {O}{(h)}, \end{aligned}$$

where $g_{k}^{(\alpha )}$ is defined in Eq. (2). Then the corresponding finite difference scheme

3 $$\frac{u_{i}^{(m)}-u_{i}^{(m-1)}}{{\varDelta }{t}}=\frac{d_{1}}{h^{\alpha }}\sum _{k=0}^{i+1}g_{k}^{(\alpha )}u_{i-k+1}^{(m)} +\frac{d_{2}}{h^{\alpha }}\sum _{k=0}^{N-i+2}g_{k}^{(\alpha )}u_{i+k-1}^{(m)}+f_{i}^{(m)}$$

is unconditionally stable, see Meerschaert and Tadjeran (2004, 2006) for details.

Let $u^{(m)}=[u_{1}^{(m)},u_{2}^{(m)},\ldots ,u_{N}^{(m)}]^{T}\in {\mathbb {R]}}^N, f^{(m)}=[f_{1}^{(m)},f_{2}^{(m)},\ldots ,f_{N}^{(m)}]^{T}\in {\mathbb {R}}^N.$ Then we can rewrite (3) into the matrix form

4 $$\left(\frac{h^{\alpha }}{{\varDelta }{t}}I-A^{(m)}\right)u^{(m)}=\frac{h^{\alpha }}{{\varDelta }{t}}u^{(m-1)}+h^{\alpha }f^{(m)},$$

with

$$A^{(m)}=d_{1}G_{\alpha }+d_{2}G_{\alpha }^{T},$$

where

$$\begin{aligned} G_{\alpha }&= \left[ \begin{array}{cccccc} g_{1}^{(\alpha )}&g_{0}^{(\alpha )}&0&\cdots &0&0\\ g_{2}^{(\alpha )}&g_{1}^{(\alpha )}&g_{0}^{(\alpha )}&0&\cdots &0\\ \vdots &g_{2}^{(\alpha )}&g_{1}^{(\alpha )}&\ddots &\ddots &\vdots \\ \vdots &\ddots &\ddots &\ddots &\ddots &0\\ g_{N-1}^{(\alpha )}&\ddots &\ddots &\ddots &g_{1}^{(\alpha )}&g_{0}^{(\alpha )}\\ g_{N}^{(\alpha )}&g_{N-1}^{(\alpha )}&\cdots &\cdots &g_{2}^{(\alpha )}&g_{1}^{(\alpha )} \end{array} \right] _{N\times N}. \end{aligned}$$

We can note that $G_{\alpha }$ is a nonsymmetric Toeplitz matrix, thus it can be stored with $N+1$ entries (Wang et al. 2010). The Toeplitz matrix-vector product (MVP) can be computed in ${\mathcal {O}}(N \log N)$ complexities with the aid of FFTs (Pang and Sun 2012).

Define $v_{N,M}=\frac{h^{\alpha }}{{\varDelta }{t}}={(x_{R}-x_{L})^{\alpha }}T^{-1}\frac{M}{{(N+1)}^{\alpha }},$ which is related to the number of time steps and grid points. The above linear system (4) can be rewritten in the following matrix form

5 $$M^{(m)}u^{(m)}=b^{(m-1)},$$

where

$$M^{(m)}=\frac{h^{\alpha }}{{\varDelta }{t}}=v_{N,M}I-A^{(m)}=v_{N,M}I-(d_{1}G_{\alpha }+d_{2}G_{\alpha }^{T}),$$

and the right hand vector

$$b^{(m-1)}=v_{N,M}(u^{(m-1)}+{\varDelta }{t}f^{(m)}).$$

In order to illustrate the convergence and stability of the implicit difference scheme (3), we note that $g_{k}^{(\alpha )}$ satisfy the following proposition.

### **Proposition 1**

(Meerschaert and Tadjeran 2004; 2006; Wang et al. 2010) *Let*$1<\alpha <2$*and*$g_{k}^{(\alpha )}$*be defined in* (2). *Then we have*

$${\left\{ \begin{array}{ll} g_{0}^{(\alpha )}=1, \ g_{1}^{(\alpha )}=-\alpha<0, \ g_{2}^{(\alpha )}>g_{3}^{(\alpha )}>\cdots >0,\\ \sum _{j=0}^{\infty }g_{j}^{(\alpha )}=0, \ \sum _{j=0}^{n}g_{j}^{(\alpha )}<0, \quad \text {for}\ n\ge 1,\\ g_{j}^{(\alpha )}={\mathcal {O}}(j^{-(\alpha +1)}). \end{array}\right. }$$

Since $$\mid {g_{1}^{(\alpha )}} \mid > \sum _{j=0,j\ne 1}^{n}g_{k}^{(\alpha )},$$$$M^{(m)}=v_{N,M}I-A^{(m)}$$ is a strongly diagonally dominant and nonsingular Toeplitz matrix, and thus the scheme (3) is monotone; refer to Wang et al. (2010).

## The BiCGT method and the BiCRT method

We will show how to construct the permutation preconditioners for accelerating the iterative solver and describe the derivation process of BiCGT and BiCRT. Furthermore, an analysis of computational cost for each iteration step is also proposed. In the linear system (5), $M^{(m)}$ is a nonsymmetric Toeplitz matrix. As previously mentioned, we cannot exploit BiCG and BiCR for resulting linear systems (5) directly, otherwise it is not possible to take advantage of the Toeplitz structure of coefficient matrix. So we need to modify and improve the BiCG and BiCR methods particularly for solving (5). Recently, Sogabe et al. (2005), proposed a preconditioner of permutation matrix for improving the performance of the Krylov subspace method, which is used to solve a nonsymmetric Toeplitz linear system. Later, Pestana and Wathen rigorously establish a circulant preconditioned MINRES method (Paige and Saunders 1975) for nonsymmetric Toeplitz systems. Inspired by their pioneer work, we construct a preconditioner, which is a permutation matrix *P* (Sogabe et al. 2005) with the form of

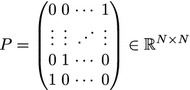



We would like to solve the Toeplitz system (5) by the CG-like method. This goal can be achieved with little additional computing cost, since we can get the equivalent system:

6 $$(PM^{(m)})u^{(m)}=Pb^{(m-1)},$$

which can be regarded as a left preconditioning technique (Saad 2003) and also has the same solution with (5). Define $$\tilde{M}^{(m)}=PM^{(m)},$$$$\tilde{b}^{(m-1)}=Pb^{(m-1)},$$ then (6) can be rewritten into

7 $$\tilde{M}^{(m)}u^{(m)}=\tilde{b}^{(m-1)},$$

an equivalent statement is that $M^{(m)}$ is self-adjoint with respect to the bilinear form defined by *P* (Paige and Saunders 1975). *P* is symmetric positive definite, a nonsymmetric Toeplitz matrix is exactly changed into a symmetric matrix $\tilde{M}^{(m)}$, so that (7) can be solved by the modified BiCG, where the additional operations for dual systems have been eliminated.

Firstly, we consider CG and BiCG (Saad 2003). Then we followed the philosophy behind the derivation of iterative method in Sogabe et al. (2005 Algorithm1), i.e., in the CG Algorithm, we replace *A* and *b* with $\tilde{A}=PM$ and $\tilde{f}=Pf$, respectively. Then we get the following new algorithm:

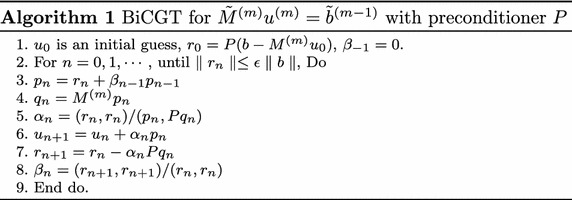



In BiCGT, we only need one MVP, i.e. $M^{(m)}p_{n},$ and two inner products, three vector additions/subtractions per iteration. The rewritten algorithm is more effective than CGNR, because *P* multiply an arbitrary vector is to reorder the vector in its reversed order (Sogabe et al. 2005). Therefore, it can greatly reduce the required number of MVPs.

In a similar way, we can get the algorithm of BiCRT for symmetric linear system (7), BiCRT reduces to CR if we get rid of the permutation preconditioner *P*, and the algorithm of BiCRT is presented as blew. 
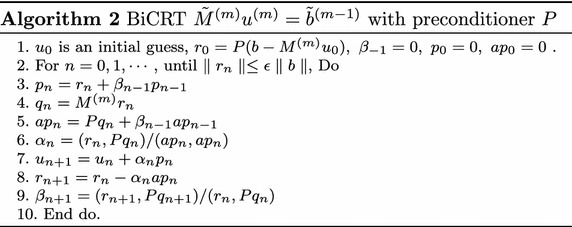


If we employ BiCG or BiCR directly, it is impossible to minimize the residual vector in some special conditions. However, BiCRT could realize this goal to some extent. The approximation ${u_{n+1}}$ is generated from ${u_{n}}$ by moving from ${u_{n}}$ in a certain direction $p_{n}$ to a minimum point of the residual function $E({u})=\Vert \tilde{M}^{(m)}u-\tilde{b}^{(m-1)}\Vert _{2}$, $u\in {\mathbb {R}}^{N}$. In other words, for ${u}_{n+1}={u}_{n}+{\alpha }_{n}{p}_{n}$, ${\alpha }_{n}$ is chosen to minimize *E*(*u*).

**Table 1:** Summary of algorithmic cost per iteration step

Method	Dot product	AXPY	MVP
BiCG	2	5	1+1
BiCR	2	6	1+1
BiCGT	2	3	1
BiCRT	2	4	1
CGNR	2	3	2

It is useful to consider the computational cost. We give a table to illustrate the computational cost of BiCG, BiCR, BiCGT, BiCRT and CGNR. “AXPY” denotes addition of scaled vectors, and “$1+1$” denotes 1 product with the matrix and 1 with its transpose. From Table 1, it is remarkable that BiCG and BiCR, BiCGT and BiCRT require almost the same memory and computational cost in each iteration step. More precisely, BiCGT is the best method to solve the above system in terms of computational cost (i.e, AXPYs and MVPs). For BiCRT, the number of AXPYs is one more than BiCGT, and for CGNR, the number of MVPs is one more than BiCGT. As we know, the computational complexity of one Toeplitz MVP is ${\mathcal {O}}{(N \log N)}$ by FFTs, but one AXPY can be computed in ${\mathcal {O}}{(N)}$ complexity. So BiCRT is more efficient than CGNR from this perspective.

## Numerical results

We solve the FDEs (1) numerically by the implicit finite difference method given in Sect. 2. After the finite difference discretization and the equivalent transformation, the symmetric linear system (7) is solved by BiCGT (Algorithm 1), BiCRT (Algorithm 2), and CGNR (Wang and Wang 2011), respectively. All the MVPs $\tilde{M}^{(m)}u^{(m)}$ are done by FFTs in ${\mathcal {O}}{(N \log N)}$ operations (Lei and Sun 2013) and the initial guess is chosen to be zero vector at each time step. The stopping criterion of BiCGT, BiCRT and CGNR is set to be

$$\frac{\Vert r^{(k)} \Vert _{2}}{\Vert r^{(0)} \Vert _{2}} < 10^{-7},$$

where $r^{(k)}$ is the residual vector of the linear system after *k* iterations and $r^{(0)}$ is the initial residual vector.

In the following tables, “*N*” denotes the number of spatial grid points, “*M*” denotes the number of time steps, CPU(s) denotes the total CPU time (in seconds) for solving the whole discretized system. “Error” denotes the infinity norm of the difference between the true solution and the approximation at the last time step. “Iter” denotes the average number of iterations over all time discretized level for solving the FDEs, i.e., Iter $=\frac{1}{M} \sum \nolimits _{m=1}^{M}$Iter (*m*), where Iter(*m*) is the number of iterations required for solving the linear system (7) in the *m*th time discretized level. All experiments are run in MATLAB R2010a on a PC with the following configuration: Windows 7 (32 bit), Iter(R) Core(TM) i3-2130 CPU 3.40 GHz and 4 GB RAM.

### *Example 1*

We consider FDEs (1) on space interval $[x_{L},x_{R}]=[0,1]$ and time interval $[0,T]=[0,1]$ with diffusion coefficients $d_{1}=0.8,\ d_{2}=0.2,$ initial condition $u_{0}(x)=\sin (1)x^{3}(1-x)^{3},$ and source term

$$\begin{aligned} f(x,t) &=\cos (t+1)x^{3}(1-x)^{3}-\sin (t+1)\Bigg \{\frac{{\varGamma }(4)}{{\varGamma }(4-\alpha )}[d_{1} x^{3-\alpha }+d_{2}(1-x)^{3-\alpha }]\\&\quad -3\frac{{\varGamma }(5)}{{\varGamma }(5-\alpha )}[d_{1}x^{4-\alpha } +d_{2}(1-x)^{4-\alpha }]+3\frac{{\varGamma }(6)}{{\varGamma }(6-\alpha )}[d_{1}x^{5-\alpha }+d_{2}(1-x)^{5-\alpha }]\\&\quad -\frac{{\varGamma }(7)}{{\varGamma }(7-\alpha )}[d_{1}x^{6-\alpha }+d_{2}(1-x)^{6-\alpha }] \Bigg \}. \end{aligned}$$

The exact solution is $u(x,t)=\sin (t+1)x^{3}(1-x)^{3}.$ For the finite difference discretization, the space step and time step are taken to be $h=1/(N+1)$, ${\varDelta }t = 2h$, i.e., $N+1=2M$.

**Fig. 1:**
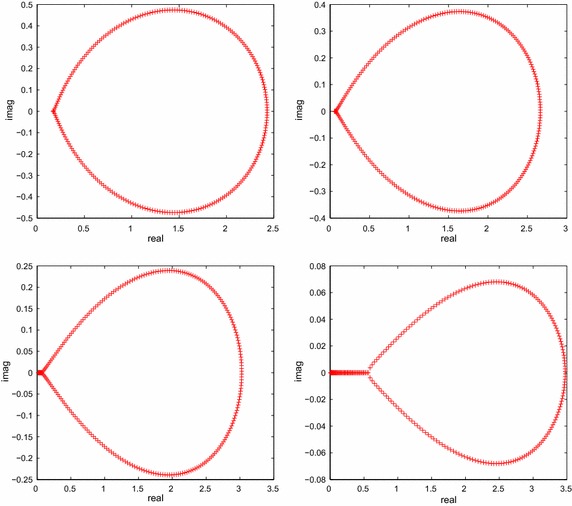
The spectra of the matrix $M^{(m)}$ with $\alpha =1.2,1.4,1.6,1.8$ for Example 1

The numerical results are listed in Table 2, as for comparisons, we also carry out CGNR without preconditioner. From Table 2, it is remarkable that the error is improved for CGNR, BiCGT and BiCRT as the increasing of $\alpha$. However, BiCGT and BiCRT are more effective than CGNR in terms of CPU time. More precisely, the performance of BiCGT is the best in terms of the CPU time except the cases of $\alpha =1.4, N=255$, $\alpha =1.4, N=511$, $\alpha =1.4, N=1023$, and $\alpha =1.8, N=1023$. In addition, the average number of iterations of BiCGT is less than that by CGNR and BiCRT sometimes. For instance, look at these cases in the numerical results at discretized size $N=127, M=64$, $N=255, M=128$, and $N=511, M=256$ for $\alpha =1.8$. BiCGT and BiCRT have faster convergence speed, less computational time expenditure than CGNR. Meanwhile, we can see that the CPU time increases as the order $\alpha$ of the time derivative increases. To explain this phenomenon, we list the spectra of the original matrix $M^{(m)}$ with different $\alpha$ in Fig. 1. As we can see from the figure, most of eigenvalues is close to zero with the increasing of the valve of $\alpha$, it means that the coefficient matrix become increasingly ill-conditioned, it also implies that the linear systems will be difficult to solve.

**Table 2:** Comparisons for solving Example 1 by different methods with $\alpha =1.4, \ 1.5$ and 1.8 at $t=1$

$\alpha$	$N+1$	CGNR			BiCGT			BiCRT		
		CPU(s)	Error	Iter	CPU(s)	Error	Iter	CPU(s)	Error	Iter
1.4	64	0.129	3.7873e−4	69.6	0.101	3.7873e–4	63.6	0.103	3.7873e–4	63.7
	128	1.038	1.9163e–4	163.1	0.632	1.9163e−4	144.0	0.646	1.9163e−4	143.7
	256	4.828	9.6389e−5	272.0	3.963	9.6389e−5	354.0	3.898	9.6390e−5	338.7
	512	28.391	4.8338e−5	375.8	25.382	4.8338e−5	590.5	23.993	4.8342e−5	546.4
	1024	136.318	2.4205e−5	476.0	129.990	2.4206e−5	838.5	114.42	2.4212e−5	726.3
1.5	64	0.140	2.7756e−4	75.7	0.107	2.7756e−4	65.4	0.150	2.7756e−4	65.4
	128	1.206	1.4046e−4	188.2	0.760	1.4046e−4	162.6	0.844	1.4046e−4	162.2
	256	7.339	7.0658e−5	410.5	4.888	7.0654e−5	421.0	5.662	7.0654e−5	418.9
	512	51.154	3.5436e−5	681.4	42.872	3.5435e−5	955.4	45.933	3.5439e−5	904.0
	1024	289.79	1.7747e−5	1015.0	267.921	1.7744e−5	1657.2	277.638	1.7751e−5	1464.7
1.8	64	0.186	8.0708e−5	103.8	0.131	8.0708e−5	85.2	0.140	8.0708e−5	85.0
	128	1.852	4.0979e−5	292.8	1.042	4.0979e−5	234.3	1.100	4.0978e−5	234.0
	256	14.583	2.0659e−5	841.5	8.570	2.0659e−5	746.9	8.629	2.0659e−5	729.4
	512	182.390	1.0375e−5	2472.5	96.153	1.0374e−5	2205.7	96.526	1.0374e−5	2164.3
	1024	1708.245	5.2057e−6	6053.9	1075.756	5.1984e−6	6778.4	1058.220	5.1985e−6	6604.0

### *Example 2*

Consider FDEs (1) on space interval $[x_{L},x_{R}]=[0,1]$ and time interval $[0,T]=[0,1]$ with diffusion coefficients $d_{1}=0.8,\ d_{2}=0.2,$ initial condition $u_{0}(x)=x^{2}(1-x)^{2},$ source term

$$\begin{aligned} f(x,t) &=-e^{-t}\Big\{x^2(1-x)^2+\frac{{\varGamma }(3)}{{\varGamma }(3-\alpha )}[d_{1}x^{2-\alpha }+d_{2}(1-x)^{2-\alpha }] \\&\quad -2\frac{{\varGamma }(4)}{{\varGamma }(4-\alpha )}[d_{1}x^{3-\alpha }+d_{2}(1-x)^{3-\alpha }] +\frac{{\varGamma }(5)}{{\varGamma }(5-\alpha )}[d_{1}x^{4-\alpha }+d_{2}(1-x)^{4-\alpha}] \Big\}. \end{aligned}$$

The exact solution of this example is $u(x,t)=e^{-t}x^2(1-x)^2.$ For the finite difference discretization, the space step and time step are taken to be $h=1/(N+1)$, ${\varDelta }t = 2h$ and $N+1=2M$, respectively.

**Fig. 2:**
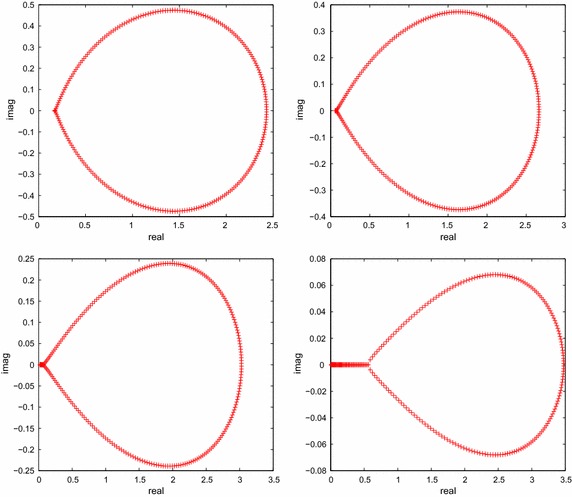
The spectra of the matrix $M^{(m)}$ with $\alpha =1.2,.4,1.6,1.8$ for Example 2

Table 3 shows the numerical results for solving Example 2 by different methods. The error is decreased for those methods as the increasing of $\alpha$, and the accuracy is almost the same as Example 1. Similar to Example 1, BiCGT and BiCRT are more effective than CGNR in terms of CPU time elapsed. Besides, the CPU time increases as the order $\alpha$ of the time derivative increases is similar to Example 1, and the reason is the same as Example 1. We also list the spectra of the matrix $M^{(m)}$ with different $\alpha$ in Fig. 2.

**Table 3:** Comparisons for solving Example 2 by different methods with $\alpha =1.4,\ 1.5$ and 1.8 at $t=1$

$\alpha$	$N+1$	CGNR			BiCGT			BiCRT		
		CPU(s)	Error	Iter	CPU(s)	Error	Iter	CPU(s)	Error	Iter
1.4	64	0.131	4.0801e−4	70	0.101	4.0801e−4	64.1	0.103	4.0801e−4	64.2
	128	1.007	2.0542e−4	157.3	0.671	2.0542e−4	151.9	0.682	2.0542e−4	151.7
	256	4.764	1.0283e−4	274.0	3.985	1.0283e−4	353.7	3.977	1.0283e−4	349.0
	512	30.947	5.1397e−5	408	27.848	5.1395e−5	643.2	26.268	5.1398e−5	601.4
	1024	148.506	2.5696e−5	519	147.36	2.5677e−5	948.6	130.875	2.5682e−5	837.8
1.5	64	0.147	2.7980e−4	76.3	0.100	2.7980e−4	66.9	0.111	2.7980e−4	66.6
	128	1.286	1.4222e−4	190.0	0.745	1.4222e−4	171.5	0.791	1.4222e−4	171.0
	256	7.410	7.1577e−5	397.5	4.806	7.1582e−5	427.2	4.962	7.1582e−5	426.0
	512	56.933	3.5883e−5	713.8	43.722	3.5877e−5	1012.6	42.825	3.5883e−5	972.4
	1024	331.186	1.7968e−5	1106	286.337	1.7953e−5	1841.4	261.763	1.7948e−5	1659.9
1.8	64	0.182	9.8722e−5	105.5	0.144	9.8722e−5	91.3	0.237	9.8722e−5	90.2
	128	1.890	4.6159e−5	298.2	1.088	4.6159e−5	241.3	1.592	4.6159e−5	240.1
	256	15.027	2.2331e−5	858.4	8.798	2.2331e−5	758.0	10.687	2.2331e−5	746.2
	512	189.009	1.0989e−5	2503.8	105.062	1.0989e−5	2332.5	133.211	2.2331e−5	2283.1
	1024	1733.04	5.4529e−6	6104.3	1158.655	5.4529e−6	7074.2	1007.543	5.4529e−6	6900.6

## Concluding remarks

Two new iterative methods, named BiCGT and BiCRT, are presented to solve the resulting linear system of the FDEs (1), which are discretized by the implicit finite difference method. Namely, with the help of the permutation matrix *P*, we transform the difficult nonsymmetric linear systems into the symmetric cases, which are often simpler to be solved. The computational complexity can be reduced from ${\mathcal {O}}(N^3)$ to ${\mathcal {O}}(N \log N)$ by utilizing FFTs. Numerical experiments illustrate the effectiveness of the proposed methods. In our future work, we will apply BiCGT and BiCRT to solve other (two dimensional) fractional differential equations (Wang and Basu 2012), such as fractional advection-diffusion equations; and we will investigate and develop some suitable preconditioning, see e.g. Lei and Sun (2013), Gu et al. (2015) to further accelerate our proposed methods.
